# Femorotibial kinematics in dogs with cranial cruciate ligament insufficiency: a three-dimensional in-vivo fluoroscopic analysis during walking

**DOI:** 10.1186/s12917-018-1395-2

**Published:** 2018-03-12

**Authors:** Selena Tinga, Stanley E. Kim, Scott A. Banks, Stephen C. Jones, Brian H. Park, Antonio Pozzi, Daniel D. Lewis

**Affiliations:** Comparative Orthopaedics and Biomechanics Laboratory, College of Veterinary Medicine, and the Department of Mechanical & Aerospace Engineering, University of Florida, PO Box 100126, 2015 SW 16th Ave, Gainesville, FL 32610-0126 USA

**Keywords:** Femorotibial, Stifle, Kinematics, Cranial cruciate ligament

## Abstract

**Background:**

Cranial cruciate ligament (CrCL) insufficiency is a degenerative condition that is a common cause of pelvic limb lameness and osteoarthritis in dogs. Surgical therapies developed to treat dogs with naturally occurring CrCL insufficiency aim to address the resultant instability, but the in-vivo alterations in stifle kinematics associated with CrCL insufficiency have not been accurately defined. The objective of this study was to quantify the 3-dimensional femorotibial joint kinematics of dogs with naturally occurring cranial cruciate ligament (CrCL) insufficiency during ambulation. Eighteen client-owned dogs (20–40 kg) with natural unilateral complete CrCL rupture were included. Computed tomographic scans were used to create digital 3-dimensional models of the femur and tibia bilaterally for each dog. Lateral fluoroscopic images were obtained during treadmill walking and 3 complete gait cycles were analyzed. Stifle flexion/extension angle, craniocaudal translation, and internal/external rotation were calculated throughout the gait cycle using a previously described 3D-to-2D image registration process. Results were compared between the pre-operative CrCL-deficient and 6-month post-operative contralateral stifles (control).

**Results:**

CrCL-deficient stifles were maintained in greater flexion throughout the gait cycle. Cranial tibial subluxation was evident in CrCL-deficient stifles at all time points throughout the gait cycle [9.7 mm at mid-stance (*P* < 0.0001); 2.1 mm at mid-swing (*P* < 0.0017)], and the magnitude of cranial tibial subluxation was greater at mid-stance phase than at mid-swing phase (P < 0.0001). Greater internal tibial rotation was present in CrCL-deficient stifles during stance phase (*P* < 0.0022) but no difference in axial rotation was evident during swing phase.

**Conclusions:**

Naturally occurring CrCL rupture causes profound craniocaudal translational and axial rotational instability, which is most pronounced during the stance phase of gait. Surgical stabilization techniques should aim to resolve both craniocaudal subluxation and axial rotational instability.

## Background

Cranial cruciate ligament (CrCL) insufficiency is a degenerative condition [1, 2] that is a common cause of pelvic limb lameness [3] and osteoarthritis [4–8] in dogs. Pet owners were estimated to have spent over $1 billion treating dogs with CrCL insufficiency in 2003 [9]. Conservative medical management options are available and may result in improved function in some dogs, particularly dogs weighing less than 15 kg [10]. Surgical management, however, is typically recommended to address joint instability, mitigate the progression of osteoarthritis, and address concurrent meniscal pathology [10, 11].

Experimental transection of the CrCL invariably leads to stifle joint instability, [5, 12–14] and has been used to study the development of osteoarthritis [4–8]. The presumed effects of CrCL insufficiency on joint motion during ambulation in-vivo are derived from 2 studies that were performed using invasive experimental techniques in normal dogs [5, 13]. Both studies measured joint kinematics before and after experimental unilateral CrCL transection by tracking metal implants (metal bone plates with removable spatial linkage [13] or implanted metal beads [5]) with biplanar radiophotogrammetry or fluoroscopy. However, acute experimental CrCL transection may not accurately replicate the pathology and biomechanics that are present in dogs with naturally occurring CrCL insufficiency. Additionally, there are logistical and ethical issues associated with the invasive nature of implanting metallic markers when studying the effects of CrCL insufficiency in client owned animals. A recent clinical study used lateral fluoroscopy to document stifle instability in a series of client owned dogs with natural CrCL insufficiency, but unfortunately the kinematic evaluation was limited to subjective qualitative assessments [15]. Recently, our group validated the use of single plane fluoroscopy (without the use of metal implants) for accurate quantification of bone motion in 3 dimensions [16] using methodology developed to study human knee kinematics [17–19]. While biplanar fluoroscopy is considered the most accurate modality for determining joint kinematics, this study demonstrated that the single plane modality was accurate to within 1.28 mm for translations and 1.58° for rotations [16].

Surgical therapies developed to treat dogs with naturally occurring CrCL insufficiency aim to address the resultant instability, [11] but the in-vivo alterations in stifle kinematics associated with CrCL insufficiency have not been accurately defined. Characterizing the kinematics of naturally diseased CrCL-deficient stifles would allow for more refined assessment of the efficacy of the currently advocated surgical stabilization techniques and guide the development of future treatment options. The objective of the current study was to quantify the 3-dimensional femorotibial joint kinematics of dogs with naturally occurring CrCL insufficiency during ambulation. We hypothesized that CrCL-deficient stifles would have increased femorotibial flexion, cranial tibial translation, and internal tibial rotation compared to each dog’s unaffected contralateral stifle throughout the gait cycle.

## Methods

Dogs presenting to the University of Florida Small Animal Hospital for CrCL insufficiency between July 2012 and March 2014 were evaluated for potential inclusion into the study. Adult non-chondrodystrophic dogs weighing between 20 to 40 kg with a history of unilateral lameness of less than 6-months duration were considered for enrollment. Inclusion was confirmed when (1) a unilateral complete CrCL rupture was diagnosed on orthopedic examination by a board certified surgeon based on cranial drawer and tibial compression tests (positive in the affected limb, negative in the contralateral limb), (2) stifle radiographs confirmed evidence of CrCL rupture in the affected stifle (stifle effusion ± osteoarthritis), and (3) complete CrCL rupture was confirmed at the time of surgery via arthroscopy or arthrotomy (surgeon preference). Dogs were excluded if concurrent clinical orthopedic disease was identified on physical examination, including palpable pain, effusion, or instability of the contralateral stifle. The study was approved by the University’s Institutional Animal Care and Use Committee and owners signed informed consent at the time of enrollment.

### Fluoroscopic image acquisition

Continuous lateral view fluoroscopic images centered on the stifle joints were acquired during treadmill walking using a ceiling-mounted fluoroscopic system with a flat panel detector.^1^ Dogs were walked at a velocity of 2.0–2.5 mph (0.8–1.1 m/s), similar to previous studies [20]. The speed of the treadmill was set within this range at a speed that allowed a natural walking cadence. Images were acquired using a pulse rate of 30 frames/s, pulse width of 1 ms, and an image area of 410 × 300 mm, giving a 0.20 mm × 0.20 mm pixel resolution. The x-ray source was initially programmed to supply a 72 kV beam with a 50 mA beam current, with slight adjustments to parameters to optimize osseous definition for each subject. Fluoroscopic imaging was obtained for approximately 15 full gait cycles with the stifles centered in the field of view. Fluoroscopic sessions were also videotaped for later review to ensure a natural cadence was present and to aid in defining stance and swing phases of gait. Three representative gait cycles were chosen for processing. Radiation-associated risk was considered negligible.

### 3-dimensional model creation

**Fig. 1:**
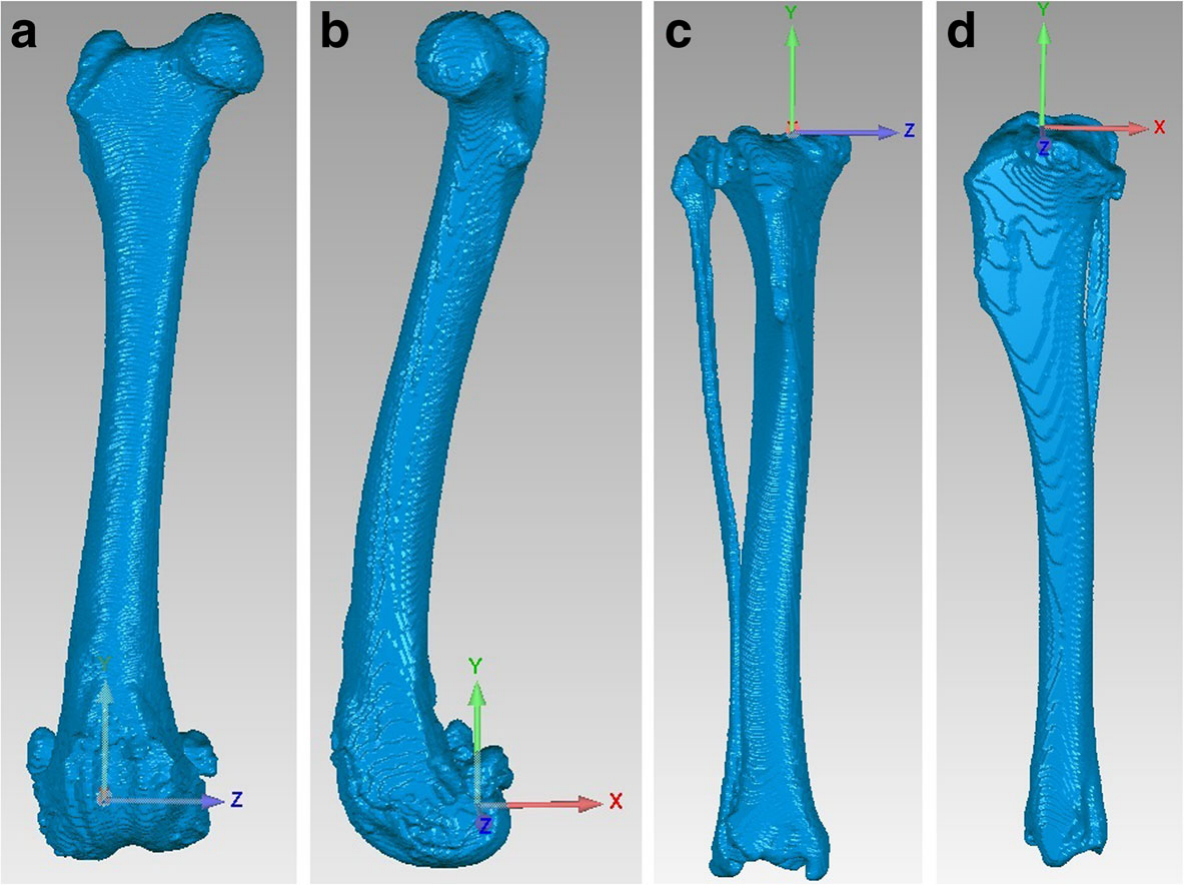
Femoral and tibial coordinate systems. CT-generated 3-dimensional digital models of the femur (craniocaudal (1**a**) and lateral (1**b**) views) and tibia (craniocaudal (1**c**) and lateral (1**d**) views) with 3-dimensional coordinate system applied

Computed tomographic (CT) scans^2^ were obtained extending from the hips through the tarsi. CT scans used a 512 × 512 image matrix, a 0.35 × 0.35 pixel dim, and 0.5 mm slice thickness with 0.3 mm overlap throughout the length of the femur and tibia. Radiation-associated risk was considered negligible. Bilateral femur/fabellae and tibia/fibula digital bone models were created using an open source 3D segmentation software program^3^ followed by a reverse engineering program.^4^ A 3-dimensional coordinate system based on anatomic landmarks was applied to each of the CT generated bone models similar to previous studies (Fig. 1) [16, 21–24]. Initially, femoral coordinates were applied such that the z-axis (mediolateral) passed through the center of the femoral condyles while remaining perpendicular to the longitudinal anatomic axis of the femur in the frontal plane. The y-axis (proximodistal) was perpendicular to the z-axis, along a plane that intersected the previously determined center of the femoral head and center of each medial and lateral femoral condyle, passing through the intercondylar notch in the frontal plane. Initially, tibial coordinates were applied such that the z-axis passed through the most prominent medial and lateral points of the tibial condyles, perpendicular to the longitudinal axis of the tibia in the frontal plane. The y-axis was perpendicular to the z-axis, along a plane that intersected the prominent medial and lateral points on the tibial condyles as well as a point mid-way between the medial and lateral malleoli. For both bones, the x-axes (craniocaudal) were determined by the right hand rule, which mandates that the 3rd axis be perpendicular to the first 2 axes. The origins of the femoral and tibial coordinate systems were then placed at the estimated center of the origin and insertion of the CrCL [21, 23].

### 3-dimensional to 2-dimensional image registration

**Fig. 2:**
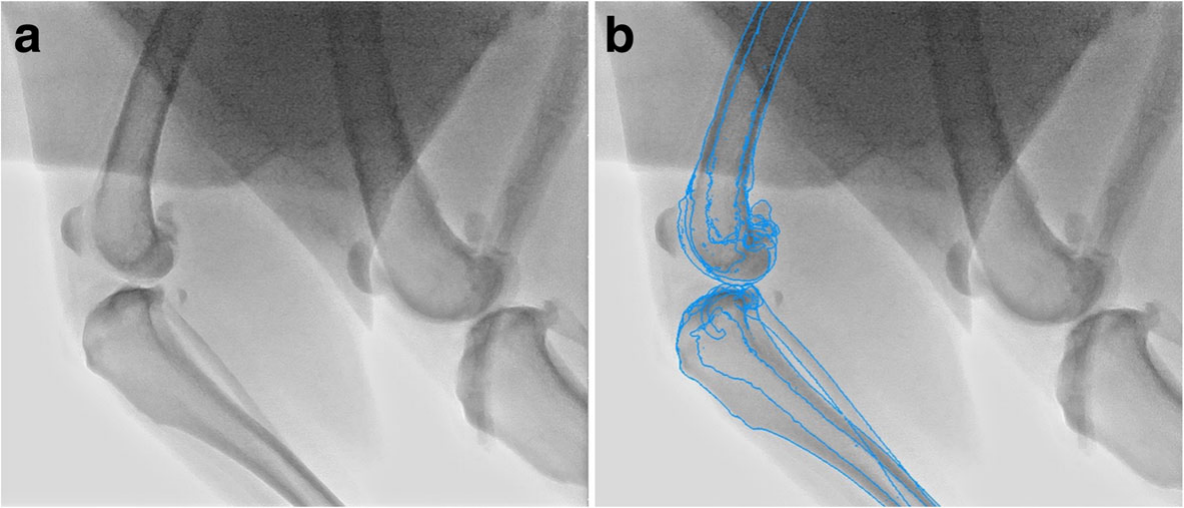
Image registration process. Fluoroscopic image before (2**a**) and after (2**b**) 3-D to 2-D image registration process. In Fig. 2b, the bone models from Fig. 1 have been projected, edge-detected, and superimposed then precisely matched to the fluoroscopic image

A previously described [16–18, 23] 3D-to-2D image registration process was used to combine 3-dimensional bone model data with 2-dimensional fluoroscopic data to ascertain 3-dimensional kinematics of the femur and tibia throughout the gait cycle (Fig. 2). The digital femur and tibia models were projected onto each frame of the fluoroscopic gait cycle, and models were manually rotated and translated until the anatomic contours of the models precisely matched the underlying image.^5^ The output of the software represents the individual model positions in space, and these results were converted to the relative positions of the bone models to each other using a custom computer program.^6^

### Control kinematic data

Contralateral limb kinematics have been shown to be affected by the presence of lameness caused by CrCL insufficiency; [25] therefore, data for the contralateral limb was collected and evaluated 6-months following tibial plateau leveling osteotomy (TPLO) of the CrCL-deficient limb. A prior study has found no difference in force plate analysis between 6-month post-operative TPLO-treated naturally affected CrCL-deficient dogs and control dogs indicating that this time frame should allow return to soundness [26].

### Kinematic data processing

The data were split into stance phase and swing phase and each phase was time normalized using a custom spline interpolation program^f^ so that a data set of 101 data points was created (or 202 data points for the complete gait cycle). Every 10th data point was chosen for statistical comparison, so that in the final data set each stance cycle had 11 data points and each swing cycle had 11 data points. This allowed averaging within and between dogs, despite temporal differences. Kinematic data was compiled for flexion-extension angle, craniocaudal translation, and internal-external rotation for both the pre-operative CrCL-deficient and the 6-month post-operative contralateral stifle (internal control). Femorotibial kinematics after TPLO treatment will be reported in a separate study.

### Statistical analysis

For each kinematic variable, 11 stance and 11 swing data points were averaged for 3 gait cycles for each dog and results were compared between affected and control stifles using a paired T-test followed by a Bonferroni correction with significance set at *P* < 0.0025. Repeated measures 2-way ANOVA was used to determine significance across the entire gait cycle for craniocaudal translation.

## Results

### Demographic information

Eighteen dogs were included in the study. Nine were mixed breed dogs, 5 were Labrador Retrievers, and the remaining dogs consisted of 1 Standard Poodle, 1 German Shepherd Dog, 1 English Springer Spaniel, and 1 Husky. Eleven dogs were spayed females and 7 were castrated males. Mean ± SD age was 6.7 ± 2.8 years. Mean ± SD body weight was 30.3 ± 5.8 kg with a median body condition score of 6/9 (range 4–8). The right stifle was affected in 10 dogs and the left in 8 dogs. Mean ± SD duration of lameness prior to presentation was 2.4 ± 2.3 months. Mean ± SD tibial plateau angle was 27.9 ± 3.0^o^ for the CrCL-deficient stifle and 28.3 ± 2.9^o^ for the contralateral control stifle (*P* = 1). On pre-operative radiographs, mild (9 dogs) to moderate (9 dogs) osteoarthritis of the affected stifle was noted. Ten dogs had no radiographic abnormalities noted in the contralateral stifle, while 8 dogs had mild osteoarthritis and effusion of the contralateral stifle detected on radiographs, despite the lack of abnormalities during clinical examination. At the time of surgery, median (range) Outerbridge scores [27] were 1 (0–3), 1 (0–2), 0.5 (0–3), and 1 (0–2) for the medial femoral condyle, medial tibial condyle, lateral femoral condyle, and lateral tibial condyle, respectively. Meniscal pathology was not identified in 8 dogs, while 10 dogs had injury to the caudal pole of the medial meniscus that required debridement. In addition to CrCL insufficiency, 5 dogs had unilateral mild to moderate osteoarthritis of the contralateral coxofemoral joint and 9 dogs had mild osteoarthritis detected in one or both tarsal joints; no pain or loss of range of motion of these joints was detected on clinical examination.

### Flexion/extension angle (Fig. 3)

Over the 11 data points during stance phase, the control stifle had mean flexion/extension angles between 137 and 147° of extension, whereas the CrCL-deficient stifle was maintained in greater flexion (*P* < 0.0007) with means between 124 and 130° of extension. Over the 11 data points during swing phase, the control stifle had means between 104 and 146° of extension; the CrCL-deficient stifle was maintained in greater flexion (*P* < 0.0001), with a means between 95 and 130° of extension.

**Fig. 3:**
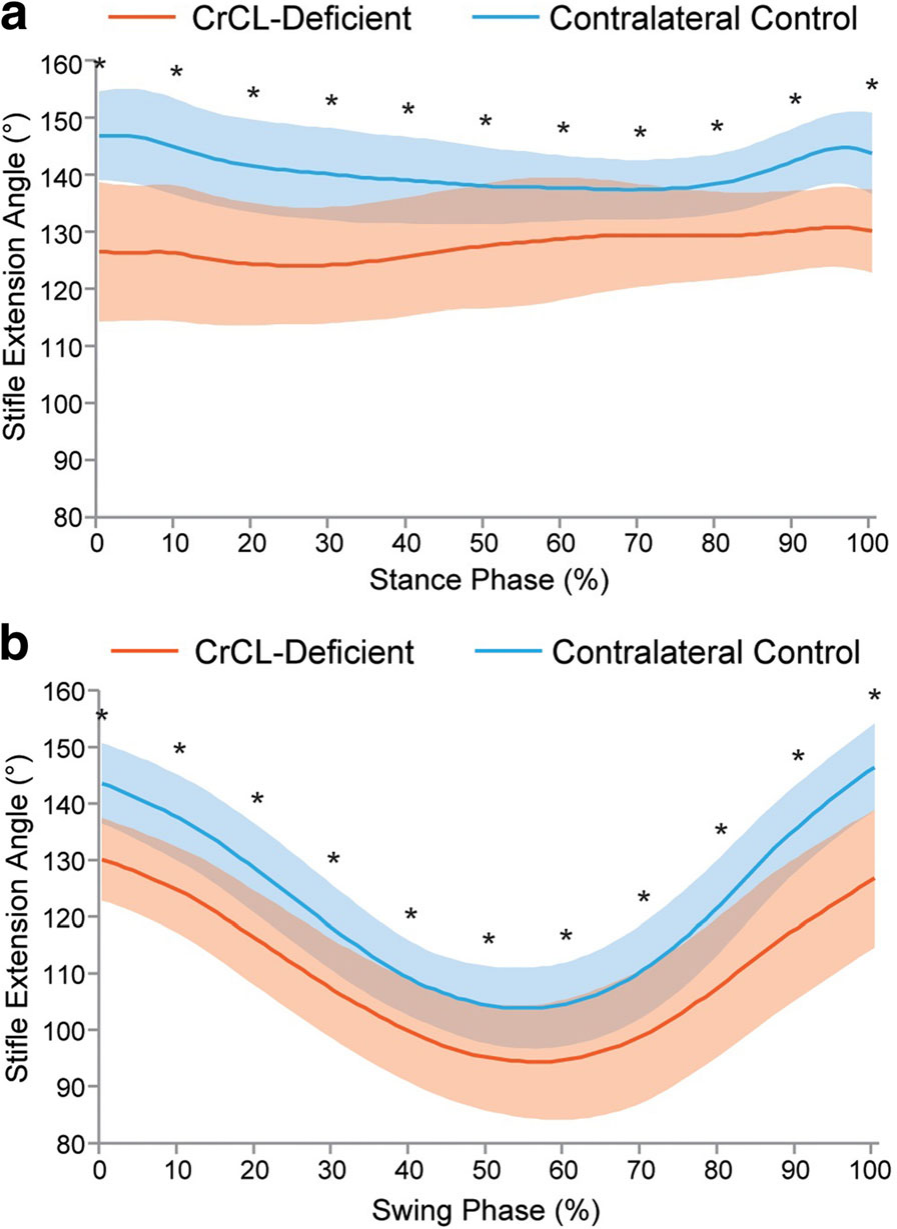
Mean flexion/extension angle during **a**) stance and **b**) swing phase in CrCL-deficient and control stifles. Orange line = CrCL-deficient, Blue line = control. Error bars represent standard deviations and * represents a statistically significant difference (using paired T-test) at that time point. The CrCL-deficient stifle was more flexed throughout the gait cycle compared to the contralateral control stifle

### Craniocaudal translation (Fig. 4)

Craniocaudal translation was evaluated by measuring the distance between the femoral origin and tibial insertion of the CrCL along the craniocaudal axis at 10% increments throughout the phases of the gait cycle. *Range of craniocaudal motion,* defined as the maximum change in craniocaudal distance between the origin and insertion of the CrCL observed throughout the gait cycle, was a mean (±SD) of 1.6 ± 0.8 mm in the control stifle and 8.6 ± 2.9 mm CrCL-deficient stifle (*P* < 0.0001).

**Fig. 4:**
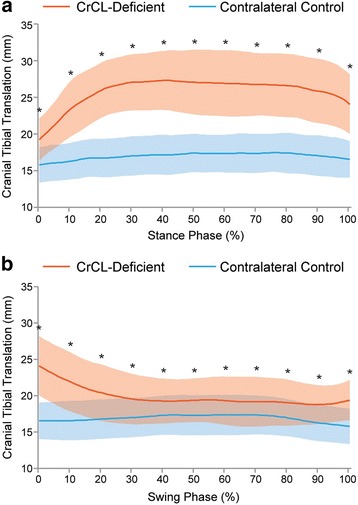
Mean cranial tibial translation during **a**) stance and **b**) swing phase in CrCL-deficient and control stifles. Orange line = CrCL-deficient, Blue line = control. Error bars represent standard deviations and * represents a statistically significant difference (using paired T-test) at that time point. The CrCL-deficient stifle had more cranial tibial translation throughout the gait cycle compared to the contralateral control stifle

*Cranial tibial subluxation* was defined as a significant difference between the CrCL-deficient and control stifles, with respect to craniocaudal distance between the origin and insertion of the CrCL at an equivalent time point during the gait cycle. In CrCL-deficient stifles, there was significant cranial tibial subluxation at all time points throughout the gait cycle (*P* < 0.0001). At mid-stance, there was 9.7 ± 2.7 mm of cranial tibial subluxation (P < 0.0001) and at mid-swing there was 2.1 ± 1.7 mm of cranial tibial subluxation (P < 0.0001). The magnitude of cranial tibial subluxation was significantly greater at mid-stance phase than at mid-swing phase (P < 0.0001).

### Internal/external rotation (Fig. 5)

Axial rotation was determined from the flexion-abduction-axial rotation ordered angle decomposition of the transformation matrix describing the tibial pose with respect to the femur [22]. This value can be thought of as the angular offset between the femoral and tibial x-axes. *Range of axial rotation* was defined as the difference between the maximum and minimum axial angular offsets throughout the gait cycle, within a joint. There was a mean (±SD) of 8.2 ± 4.4° of axial rotation range of motion in the control stifle and 8.0 ± 6.2° of axial rotation range of motion in the CrCL-deficient stifle throughout the gait cycle (*P* = 0.1085). While the range of axial rotation was similar between control and CrCL-deficient stifles, the timing of rotation differed between limbs. Both the control and CrCL-deficient stifles were maximally externally rotated in early stance phase; however, the control stifles reached maximal internal rotation at mid-swing phase and the CrCL-deficient stifles reached maximal internal rotation at mid-stance phase.

**Fig. 5:**
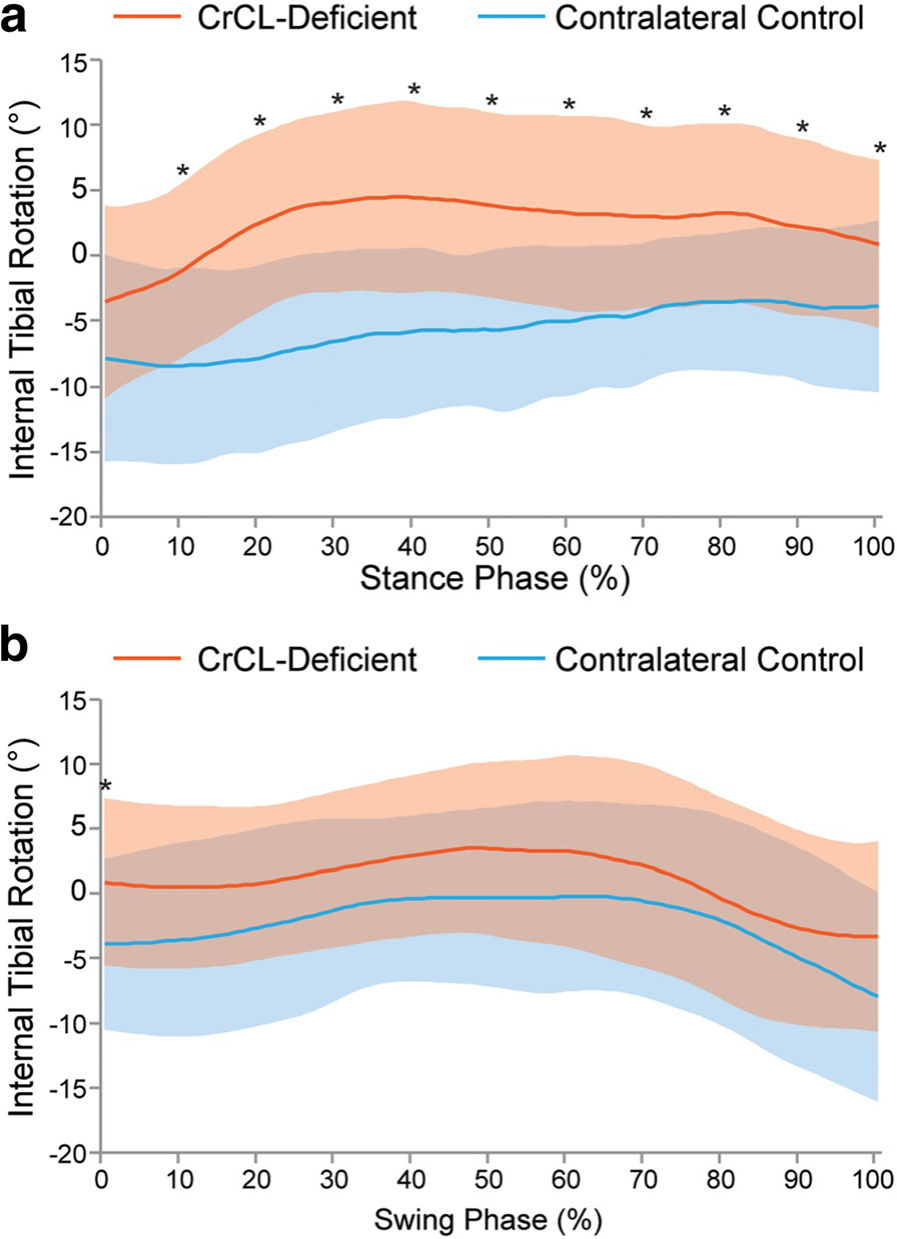
Mean internal/external rotation during **a**) stance and **b**) swing phase in CrCL-deficient and control stifles. Orange line = CrCL-deficient, Blue line = control. Error bars represent standard deviations and * represents a statistically significant difference (using paired t-test) at that time point. The CrCL-deficient stifle had more internal tibial rotation throughout stance phase compared to the contralateral control stifle and there was no difference during swing phase

*Abnormal axial rotation* was defined as a significant difference between the CrCL-deficient and control stifles, with respect to degree of axial rotation at a given equivalent time point during the gait cycle. In the CrCL-deficient stifles, the tibia was abnormally internally rotated for the majority of stance phase (*P* < 0.0022 between 10 and 100% stance phase). During the swing phase, there was no significant difference in axial rotational position between limbs.

## Discussion

The objective of this study was to quantitatively define the 3-dimensional stifle motion in dogs with naturally occurring CrCL insufficiency. We found that flexion-extension angle and craniocaudal translation were abnormal throughout the walking gait cycle, and internal-external rotation was abnormal during the stance phase. We confirmed that naturally occurring CrCL insufficiency results in profound disturbance of stifle kinematics in dogs.

CrCL-deficient stifles were maintained in 8–20° greater flexion throughout the gait cycle when compared to control stifles. Prior in-vivo studies using optical motion capture [25, 28] and biplanar fluoroscopic 3D-2D image registration [5, 13] techniques have reported a similar magnitude of increased flexion in CrCL-deficient joints, ranging from 5 to 15° [5, 13, 25, 28]. Increased stifle flexion has been ascribed to joint effusion and pain, [29, 30] both of which are present in naturally occurring and experimentally induced disease states. Increased stifle flexion may also mitigate the magnitude of cranial tibial subluxation, as the angle formed between the patellar tendon and the femorotibial joint line decreases [31, 32]. Higher stifle flexion may be the result of a change in activity of the quadriceps, gastrocnemius, or hamstrings, or a combination of changes in activity of all 3 muscles. In humans with anterior cruciate ligament insufficiency, a proportion of the population (“copers”) is able to stabilize the knee by altered muscular forces across the joint [33]. However, in contrast to human copers, dogs in our study were not able to completely overcome the cranial tibial subluxation despite increased stifle flexion.

The maximal magnitude of cranial tibial subluxation observed in our study was 9.7 mm, which occurred during the mid-stance phase. The mid-stance phase timing of maximal cranial tibial subluxation may be due to quadriceps and gastrocnemius muscle activity, which are required to support weight bearing during the stance phase [34, 35] in addition to maintaining joint extension, these muscles also exert a cranial force on the tibia (quadriceps) and caudal force on the femur (gastrocnemius), which may promote cranial tibial thrust [36]. Tashman, et al. reported a similar magnitude of subluxation (10 mm) in the in-vivo study of experimental CrCL transection, but Korvick, et al. reported a larger magnitude of subluxation (17 mm) in an earlier in-vivo study of experimental CrCL transection [5, 13]. We suspect that numerous factors could influence the maximal magnitude of cranial tibial subluxation, such as the degree of periarticular fibrosis, differences in study methodology (such as landmark identification), and dog size, breed, and activity level (e.g. type and speed of gait). Nevertheless, our results suggest that stifles with naturally occurring complete CrCL rupture have a comparable degree of cranial tibial subluxation to normal stifles subjected to experimental CrCL transection.

Despite the previous thought that the stability of the dog stifle is independent of the CrCL during stifle flexion, [12, 13, 31] mild cranial tibial subluxation was still present in CrCL-deficient stifles during the swing phase (when the stifle is in greater flexion). A similar phenomenon was found in the study by Tashman, et al., in which persistent cranial tibial subluxation was shown to develop over the 2 years following experimental CrCL transection [5]. The presence of persistent cranial tibial subluxation may be a reflection of a chronically thickened stifle that is unable to return to a completely reduced position. Furthermore, maximal hock flexion has been shown to occur during mid-swing phase, which may promote cranial tibial subluxation through increased tension on the gastrocnemius muscle [28]. Chronic CrCL insufficiency may also be associated with disruption of the balance of muscular forces (particularly quadriceps, hamstring, and gastrocnemius muscles), [37] meniscal degeneration, [38] and changes to the osseous anatomy of the joint [4, 13]. We suspect that some or all of these changes contribute to persistent cranial tibial subluxation during the swing phase, despite this being a “CrCL-independent phase” [12, 13, 31].

Stifles with CrCL insufficiency had significantly greater internal tibial rotation when compared to control stifles, occurring maximally at the mid-stance phase and therefore coinciding with maximal cranial tibial subluxation, consistent with both in-vivo [13] and ex-vivo [14, 39] studies. After CrCL loss, the collateral ligaments become the primary restraint against cranial tibial subluxation and because the lateral collateral ligament is not as taut as the medial collateral ligament in extension, [40] the lateral aspect of the tibial plateau has more latitude to translate cranially than the medial aspect of the plateau. The medial meniscus has been demonstrated to aid in resisting cranial tibial subluxation in CrCL-deficient stifles in canine cadavers, which could provide more craniocaudal stability to the medial compartment compared to the lateral compartment [41]. The differences in function between medial and lateral collateral ligaments and medial and lateral menisci likely contribute to the internal tibial rotation that occurs as the tibia translates cranially during stance phase in the absence of the CrCL [39, 40]. Surprisingly, the in-vivo study by Tashman, et al. did not report a difference in rotational alignment after CrCL transection [5]. The authors of this study postulated that bony geometry, muscular forces, or other soft tissue constraints were able to overcome the expected rotational laxity [5]. The cause of discrepant findings for axial rotational motion across in-vivo studies is unknown, but may be related to breed and conformational differences between study populations. Nevertheless, the prominence of rotational instability in dogs with CrCL insufficiency found in the current study supports clinical concerns that rotational instability may also need to be addressed during surgical treatment of CrCL insufficiency [42, 43].

There are several limitations associated with this investigation. We had narrow selection criteria based on body weight and obvious palpable stifle laxity; therefore, our results cannot be extrapolated to other populations such as small or giant breed dogs, chondrodystrophic dogs, dogs with excessive tibial plateau angles, dogs with partial CrCL tears, dogs with severe osteoarthritis, or dogs with purely traumatic CrCL ruptures. Additionally, data were collected at a single and likely variable time point from the onset of disease and thus we are unable to provide a definitive understanding of the temporal changes associated with the course of CrCL degeneration. Fluoroscopic imaging was obtained while dogs walked on a treadmill, which has been shown to result in slight variations in joint kinematics in dogs when compared to over-ground walking [44]. We did not assess activities other than walking, such as trotting or stair climbing, [23] which may have shown different results. We utilized a single-plane fluoroscopic technique, which is less precise than biplanar techniques and precludes the ability to accurately quantify translation in the mediolateral plane [17]. During modeling, there is subjectivity in coordinate assignation as well as determination of the stance and swing phases of the gait cycle; a single researcher (ST) performed these tasks to limit variability. We also recognize that stifle motion is a complex action and stifle stability is not controlled solely by the CrCL; additionally, in the face of CrCL deficiency there are likely multiple concurrent (primary or secondary) neuromuscular changes that could also affect our measured kinematic results.

Limitations also include the fact that our control data was collected from the contralateral limb 6-months following surgical treatment for CrCL insufficiency of the affected limb. The 6-month time point was chosen to mitigate the effects of lameness on contralateral limb kinematics; [26] however, the contralateral stifles may not have been normal themselves. The development of CrCL insufficiency is multifactorial [45] with one primary contributing factor being abnormal mechanical stresses secondary to variations from normal anatomy [46] and CrCL insufficiency is a bilateral disease in approximately 50% of dogs affected [47–51]. Despite some dogs having early (stable) contralateral CrCL disease, we considered the contralateral stifle to be a superior kinematic control than the alternative of using a separate population of dogs with normal stifles. Given that there was no lameness, pain, loss of range of motion, or instability associated with any joint other than the studied CrCL-deficient stifle at the time of initial and follow-up orthopedic examinations, we considered the influence of very early contralateral CrCL disease or concurrent hip or hock osteoarthritis would likely be minimal.

## Conclusions

The femorotibial kinematic changes observed in dogs with naturally occurring CrCL insufficiency were largely consistent with previous experimental studies: we observed profound craniocaudal translational and axial rotational instability that was most pronounced during the stance phase of gait. Our investigation has provided an accurate, quantitative characterization of the instability that occurs with CrCL insufficiency. Based on our results, current surgical stabilization techniques should aim to address both craniocaudal translational and axial rotational instability with hopes to slow the progression of osteoarthritis and mitigate the likelihood of post-operative meniscal damage.

## Abbreviations

CrCL: Cranial cruciate ligament
TPLO: Tibial plateau leveling osteotomy

## Footnotes

1 Toshiba American Medical Systems, Inc., Tustin, CA.

2 Toshiba Aquilon 8, Toshiba American Medical Systems, Inc., Tustin, CA.

3 ITK-SNAP, https://sourceforge.net/projects/itk-snap/files/.

4 Geomagic, Inc., Research Triangle Park, NC.

5 JointTrack, University of Florida: http://sourceforge.net/projects/jointtrack/.

6 MATLAB and Statistics Toolbox Release R2015a, The MathWorks, Inc., Natick, MA.
